# Chemotherapy-Associated Liver Injury in Patients with Colorectal Liver Metastases: A Systematic Review and Meta-analysis

**DOI:** 10.1245/s10434-012-2438-8

**Published:** 2012-07-06

**Authors:** Stuart M. Robinson, Colin H. Wilson, Alastair D. Burt, Derek M. Manas, Steven A. White

**Affiliations:** 1 Department of HPB Surgery, Freeman Hospital, Newcastle upon Tyne, UK; 2 Institute of Cellular Medicine, Newcastle University, Newcastle upon Tyne, UK; 3 Department of Cellular Pathology, Royal Victoria Infirmary, Newcastle upon Tyne, UK

## Abstract

**Background:**

Chemotherapy-associated liver injury is a major cause for concern when treating patients with colorectal liver metastases. The aim of this review was to determine the pathological effect of specific chemotherapy regimens on the hepatic parenchyma as well as on surgical morbidity, mortality and overall survival.

**Methods:**

A systematic review of the published literature and a meta-analysis were performed. For each of the variables under consideration, the effects of different chemotherapy regimens were determined by calculation of relative risks by a random-effects model.

**Results:**

Hepatic parenchymal injury is regimen specific, with oxaliplatin-based regimens being associated with grade 2 or greater sinusoidal injury (number needed to harm 8; 95 % confidence interval [CI] 6.4–13.6), whereas irinotecan-based regimens associated with steatohepatitis (number needed to harm 12; 95 % CI 7.8–26). The use of bevacizumab alongside FOLFOX reduces the risk of grade 2 or greater sinusoidal injury (relative risk 0.34; 95 % CI 0.15–0.75).

**Conclusions:**

Chemotherapy before resection of colorectal liver metastases is associated with an increased risk of regimen-specific liver injury. This liver injury may have implications for the functional reserve of the liver for patients undergoing major hepatectomy for colorectal liver metastases.

In 2008, the incidence of colorectal cancer within Europe was estimated to be 436,000, with 212,000 deaths directly attributed to this disease.^1^ Fifty percent of patients with a primary colorectal tumor will go on to develop metastatic disease in the liver, and in 25 % of patients, this is present at the time of diagnosis.^2^^–^ 4 In patients with liver-only metastases, the gold standard of treatment is liver resection, the aim of which is to remove all metastatic disease. When this is achieved, overall 5-year survival rates in the order of 50–60 % have been reported, compared to 19.5 % for patients in whom this is not possible.^5^,^6^ However, for those patients with inoperable disease, the mainstay of treatment remains systemic chemotherapy in conjunction with recent additions such as radioembolization and the more established ablative (e.g., microwave and radiofrequency ablation) therapies.^7^^–^ 9 The advent of modern chemotherapeutics such as oxaliplatin and irinotecan, as well as biological treatments such as bevacizumab (anti-vascular endothelial growth factor A [VEGF-A]) and cetuximab (anti-epidermal growth factor receptor [EGFR]), have seen median survival rates in patients with inoperable metastatic colorectal cancer entered into phase III trials rise from 6 to 12 months in the mid-1990s to 18–24 months in the latter part of the last decade.^6^

It is increasingly recognized that in those patients with initially inoperable liver metastases, chemotherapy can be effectively provided to downstage disease such that a potentially curative resection can be offered.^10^^–^ 12 This strategy, referred to as conversion chemotherapy, is a major reason for the yearly increase in the number of liver resections being performed for colorectal liver metastases.^6^ The 5-year survival in patients whose disease is successfully downstaged and who undergo subsequent surgical resection is in the order of 30 %.^13^ In addition, there is some evidence emerging that the routine use of perioperative chemotherapy, even in patients with initially operable disease, may improve long-term survival after surgery.^14^ Together, this means that an ever-increasing number of patients undergoing liver resection to treat colorectal liver metastases will have received some form of preoperative chemotherapy.

Modern chemotherapy regimens used in the management of metastatic colorectal cancer use traditional 5-fluorouracil (5-FU) and folinic acid in combination with either oxaliplatin or irinotecan. Often 5-FU, which is provided parenterally, is substituted by its oral prodrug, capecitabine.^15^ In recent years, monoclonal antibodies directed against VEGF-A (bevacizumab) and EGFR (cetuximab, panitumumab) have also been provided in an attempt to improve tumor response rates.^5^

Many observational studies have been published claiming that the use of chemotherapy before surgery can lead to injury to the hepatic parenchyma. This injury has been reported to take the form of hepatic steatosis, steatohepatitis, nodular regenerative hyperplasia, and sinusoidal obstruction syndrome, and there are numerous reviews on this subject.^16^^–^ 18 Nonetheless, a meta-analysis has never been performed.

The most effective chemotherapy strategy in patients with inoperable colorectal liver metastases is one that provides maximal disease downstaging while having a minimal effect on the non-tumor-bearing liver, subsequently reducing surgical morbidity and mortality. Similarly, in the neoadjuvant setting, the aim should be to minimize the risk of postoperative recurrence without increasing the risk associated with that operation. At the present time, however, the true magnitude of the effect of chemotherapy on the hepatic parenchyma and its subsequent effect on surgical morbidity and mortality remains ill defined because of the heterogeneous nature of published case series.

The aim of this review was to determine what the effect of specific chemotherapy regimens is on the hepatic parenchyma.

## Methods

### Literature Search

A systematic search for reports published between January 1, 1996, and June 31, 2011, was performed on Medline, Embase, and the Cochrane Library. Searches included the keywords “liver resection,” “hepatectomy,” “chemotherapy,” “steatosis,” “steatohepatitis,” and “sinusoidal obstruction syndrome.” In addition, the MeSH headings “surgical procedures, operative,” “colorectal neoplasms,” “hepatectomy,” “drug-induced liver injury,” and “fatty liver” were used. In addition to these database searches, the reference lists of review articles were hand-searched to identify further reports.

### Screening of Identified Reports

**Fig. 1:**
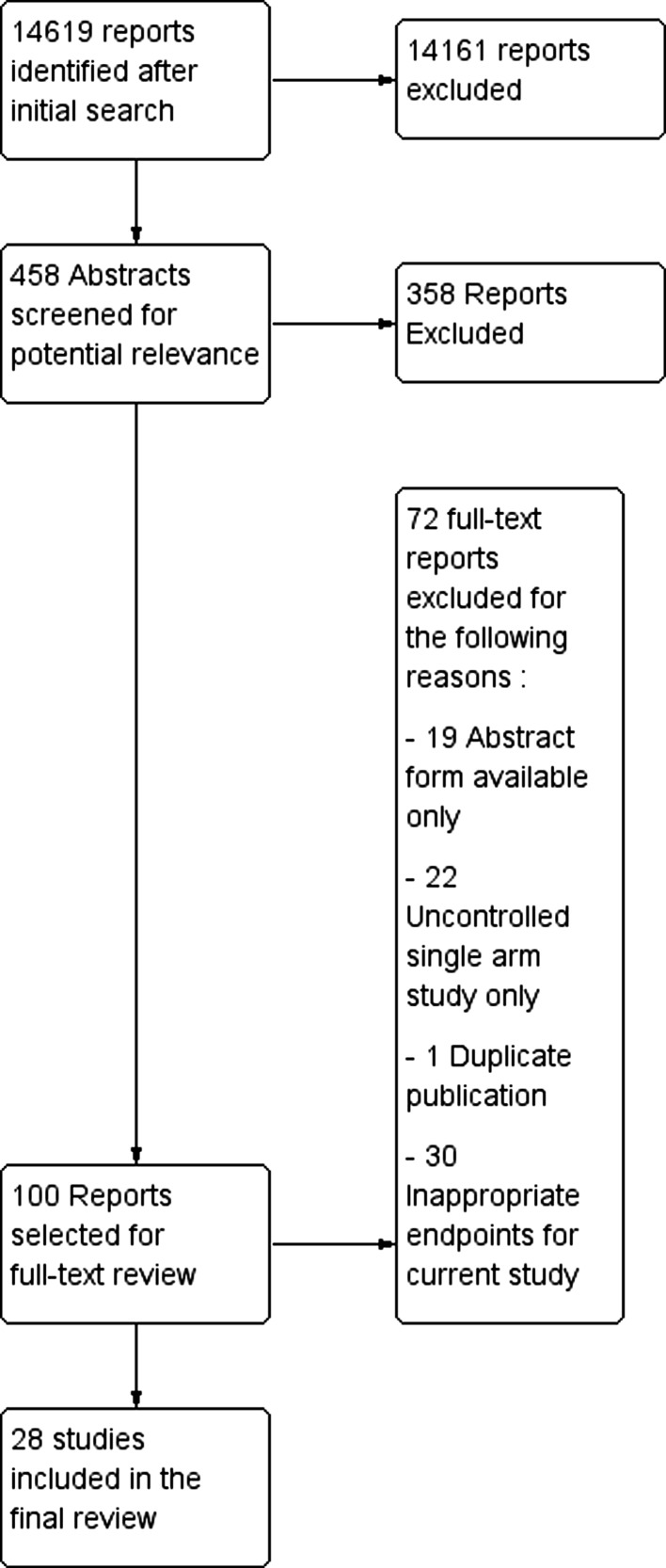
Summary of study selection process

Initially the titles of potentially eligible studies were screened and case reports, commentaries/editorials, reviews, animal studies, in vitro studies, and non-English studies were rejected. At the next stage, abstracts of the remaining studies were retrieved and reviewed for potential relevance. The full text of articles whose abstracts were identified as being of potential relevance were then retrieved and assessed against the following inclusion criteria, using a standard pro forma: those included were patients undergoing treatment of colorectal liver metastases only; either histological or outcome data were provided for patients undergoing resection of colorectal liver metastases; and there were a minimum of 10 patients per group. Studies in the format of a published abstract were excluded. Figure 1 summarizes the process of study selection.

### Data Extraction

Data including study design characteristics, histological scoring of the liver parenchyma, perioperative morbidity, and mortality were extracted for each study. Close attention was paid to the kin relationship of studies—that is, multiple publications that use the same patient cohorts. Where there was potential duplication of data, only the study that provided the largest patient number to assess a given outcome was used. Study quality was assessed according to the Newcastle–Ottawa score for nonrandomized studies.^19^ The level of evidence for each study was scored according to the Oxford Centre for Evidence Based Medicine scale.^20^ All data extraction from original articles was performed on two separate occasions and cross-referenced to ensure accuracy.

The presence of liver injury was defined according to histological criteria as follows: hepatic steatosis (the presence of either macro- or microvesicular steatosis of all grades); steatosis >30 % (hepatic steatosis involving more than 30 % of hepatocytes); steatohepatitis (the presence of the combination of steatohepatitis, inflammatory infiltrates, and ballooning degeneration of hepatocytes as assessed by a recognized scoring system such as that by Kleiner et al.^21^); and sinusoidal dilatation (dilatation of the hepatic sinusoids graded according the method of Rubbia-Brandt et al.^22^).

### Statistical Analysis

A meta-analysis was performed by using histological scoring as the outcome measures in accordance with the Meta-analysis of Observational Studies in Epidemiology Guidelines.^23^ Analysis was performed by Review Manager (RevMan) software, version 5.1 (Nordic Cochrane Centre, Copenhagen, Denmark). The effects of preoperative chemotherapy regimens on histological scores of liver injury and perioperative outcome were estimated by using a pooled relative risk (RR) along with 95 % confidence interval using a random-effects model.^24^ Studies were weighted according to population size. Overall effect size was determined with the *Z* test and statistical significance set at a level of *p* = 0.05. Heterogeneity across studies was assessed with the *I* ^2^ statistic and through forest plot inspection. Data from observational studies and randomized, controlled studies were not included within the same analysis.

## Results

### Description of Studies

Of the 14,619 reports identified within the initial search, 28 were considered appropriate for inclusion within this systematic review. Of these studies, most were considered to be of evidence level 2b or greater (*n* = 26; 93 %). All but one of the included studies were observational in character. The potential for overlap of participants was noted in 15 (54 %) of the 28 included studies. The characteristics of these studies, along with their key findings, are summarized in Table 1.

**Table 1:** Summary of included studies

Study	Years	Study type	Comparisons	*n*	NOS	Evidence level	Key findings	Overlap with other studies
Adam et al.^67^	2010	CS(R)	Neoadjuvant chemotherapy versus surgery alone	1471	7	2b	The use of preoperative chemotherapy does not seem to offer any benefit to patients with a solitary metachronous colorectal liver metastases	Data from LiverMet survey (i.e., multiple centers)
Aloia et al.^68^	2006	CS(R)	Neoadjuvant (Ox based) chemotherapy versus surgery alone	75	8	2b	The main hepatic injury after Ox-based chemotherapy is vascular not steatosis. The risk of complications is related to the duration of chemotherapy	
Aloysius et al.^69^	2007	CC(R)	Neoadjuvant (FOLFOX-4) chemotherapy versus surgery alone	50	7	3b	The use of neoadjuvant FOLFOX-4 is associated with hepatic steatosis and sinusoidal dilatation	
Nordlinger et al.^14^	2008	RCT	Perioperative (FOLFOX) chemotherapy versus surgery alone	364		1b	Preoperative FOLFOX-4 chemotherapy increases the risk of perioperative complications but improves progression free survival	Multicenter RCT
Gomez et al.^52^	2007	CS(R)	Hepatic steatosis versus no hepatic steatosis	386	8	2b	Hepatic steatosis increases the morbidity of liver resection	70
Gomez-Ramirez et al.^30^	2010	CS(P)	Neoadjuvant chemotherapy versus surgery alone	45	6	2b	Neoadjuvant irinotecan is associated with an increased risk of steatohepatitis	
Hewes et al.^71^	2007	CS(R)	Neoadjuvant chemotherapy versus surgery alone	67	8	2b	Neoadjuvant Ox-based chemotherapy increases the risk associated with liver resection	
Hubert et al.^72^	2010	CS(R)	Neoadjuvant chemotherapy versus surgery alone	114	8	2b	Neoadjuvant chemotherapy is associated with sinusoidal congestion but has no impact on perioperative outcome	
Kandutsch et al.^73^	2008	CS(R)	Neoadjuvant (Ox based) chemotherapy versus surgery alone	63	8	2b	Sinusoidal obstruction but not steatohepatitis occurs as a consequence of Ox-based chemotherapy	40 ^,^ 45
Karoui et al.^74^	2006	CS(R)	Neoadjuvant chemotherapy versus surgery alone	67	7	2b	Prolonged chemotherapy injures the hepatic parenchyma and increases the morbidity of liver resection when performed under total vascular exclusion	
Kishi et al.^75^	2010	CS(R)	Neoadjuvant FOLFOX versus neoadjuvant FOLFOX and bevacizumab	219	8	2b	Extended preoperative chemotherapy increases the risk of parenchymal injury without improving pathological response	33 ^,^ 44 ^,^ 46 ^,^ 76
Klinger et al.^45^	2009	CS(R)	Neoadjuvant (Ox based) chemotherapy versus neoadjuvant (Ox based) chemotherapy and bevacizumab	99	7	2b	Bevacizumab protects against sinusoidal obstruction syndrome but does not improve tumor response to Ox-based chemotherapy	40 ^,^ 73
Komori et al.^42^	2010	CS(R)	Neoadjuvant (FOLFOX) chemotherapy versus surgery alone	27	8	2b	FOLFOX use results in parenchymal injury but has no effect on perioperative morbidity and mortality	
Makowiec et al.^41^	2011	CS(R)	Neoadjuvant chemotherapy versus surgery alone	102	7	2b	Neither preoperative chemotherapy or the presence of parenchymal injury affect perioperative outcome	
Mehta et al.^26^	2008	CS(R)	Neoadjuvant chemotherapy versus surgery alone	173	6	2b	Ox-based chemotherapy is associated with a vascular injury to the liver parenchyma but this has no effect on perioperative outcome	
Nakano et al.^39^	2008	CS(R)	Neoadjuvant (Ox based) chemotherapy versus neoadjuvant (other regimens) chemotherapy	90	8	2b	Ox-based chemotherapy is associated with an increased incidence of sinusoidal injury. Sinusoidal injury is associated with a poorer outcome after major hepatectomy	25
O’Rourke et al.^77^	2009	CS(P)	Neoadjuvant chemotherapy versus surgery alone	37	8	2b	Liver specific MRI can accurately predict the severity of parenchymal injury	78
Ouaissi et al.^79^	2006	CS(R)	Neoadjuvant chemotherapy versus surgery alone	40	6	2b	Preoperative chemotherapy does not influence the outcome of liver resection	
Pawlik et al.^31^	2007	CS(R)	Neoadjuvant chemotherapy versus surgery alone	212	8	2b	Neoadjuvant chemotherapy is associated with parenchymal injury in 20–30 % of patients. The nature of the injury is regimen specific	
Ribero et al.^46^	2007	CS(R)	Neoadjuvant (Ox based) chemotherapy versus neoadjuvant (Ox based) chemotherapy and bevacizumab	105	8	2b	The addition of bevacizumab to Ox-based chemotherapy reduces the incidence of sinusoidal injury and increases tumor response to chemotherapy as assessed histologically	33 ^,^ 44 ^,^ 75 ^,^ 76
Rubbia-Brandt et al.^22^	2004	CS(R)	Neoadjuvant chemotherapy versus surgery alone	153	6	2b	Neoadjuvant Ox-based chemotherapy is associated with sinusoidal obstruction syndrome	44
Rubbia-Brandt et al.^44^	2010	CS(R)	Neoadjuvant (Ox based) chemotherapy versus neoadjuvant (Ox based) chemotherapy and bevacizumab versus surgery alone	385	6	2b	Ox-based chemotherapy is associated with sinusoidal obstruction syndrome, the incidence of which is reduced if provided alongside bevacizumab	22 ^,^ 33 ^,^ 46 ^,^ 75 ^,^ 76
Ryan et al.^32^	2010	CS(R)	Neoadjuvant chemotherapy versus surgery alone	334	8	2b	Neoadjuvant chemotherapy is associated with a vascular injury to the hepatic parenchyma but not steatohepatitis	80
Sahajpal et al.^80^	2007	CS(R)	Neoadjuvant chemotherapy versus surgery alone	96	7	2b	Neoadjuvant chemotherapy does not affect short term outcomes after liver resection	32
Scoggins et al.^81^	2008	CS(R)	Neoadjuvant chemotherapy versus surgery alone	186	8	2b	Neoadjuvant chemotherapy does not affect the morbidity associated with liver resection	
Tamandl et al.^40^	2011	CS(R)	Neoadjuvant chemotherapy versus surgery alone	196	8	2b	Ox-induced sinusoidal obstruction is associated with poorer overall and disease specific survival	45 ^,^ 73
Vauthey et al.^33^	2006	CS(R)	Neoadjuvant chemotherapy versus surgery alone	406	8	2b	Neoadjuvant irinotecan-based chemotherapy is associated with the development of steatohepatitis	44 ^,^ 46 ^,^ 75 ^,^ 76
Yebidela et al.^82^	2005	CC(R)	Neoadjuvant chemotherapy versus surgery alone	64	8	3b	Neoadjuvant chemotherapy does not increase surgical morbidity or mortality	

*Ox* oxaliplatin, *NOS* Newcastle Ottawa Score, *CS* cohort study, *CC* case controlled study, *RCT* randomised controlled trial, *(R)* retrospective, *(P)* prospective

### Hepatic Steatosis/Steatohepatitis

Nonalcoholic fatty liver disease exists as a spectrum of pathological changes in the hepatic parenchyma, progressing from simple steatosis to steatohepatitis and eventually hepatic fibrosis and cirrhosis. The severity of hepatic steatosis is determined by the proportion of involved hepatocytes as judged by histological review of hematoxylin and eosin-stained sections of the liver. A variety of grading systems exist, although the most commonly used is that proposed by Kleiner et al.,^21^ which classifies steatosis as absent (<5 % hepatocytes), mild (5–33 % hepatocytes), moderate (>33–66 % hepatocytes), and severe (>66 % of hepatocytes). This grading system is not uniform with others, using a cutoff of 30 and 60 % to define moderate and severe steatosis, respectively.^25^^,^ 26 Given the inherent interobserver variability in assessing steatosis, the minor differences in these grading systems are unlikely to be significant, and as such, a cutoff of 30 or 33 % was considered to be equivalent for the purposes of this analysis.^27^,^28^

The importance of hepatic steatosis in patients undergoing liver resection was demonstrated in a meta-analysis by de Meijer et al., which showed its presence to be a risk factor for increased perioperative morbidity and mortality in patients undergoing major hepatic resection (>three Couinaud segments). In patients with steatosis >30 %, the risk of death after major resection increased nearly threefold, and as such, this was the cutoff we used to identify patients with high-risk steatosis.^29^

**Fig. 2:**
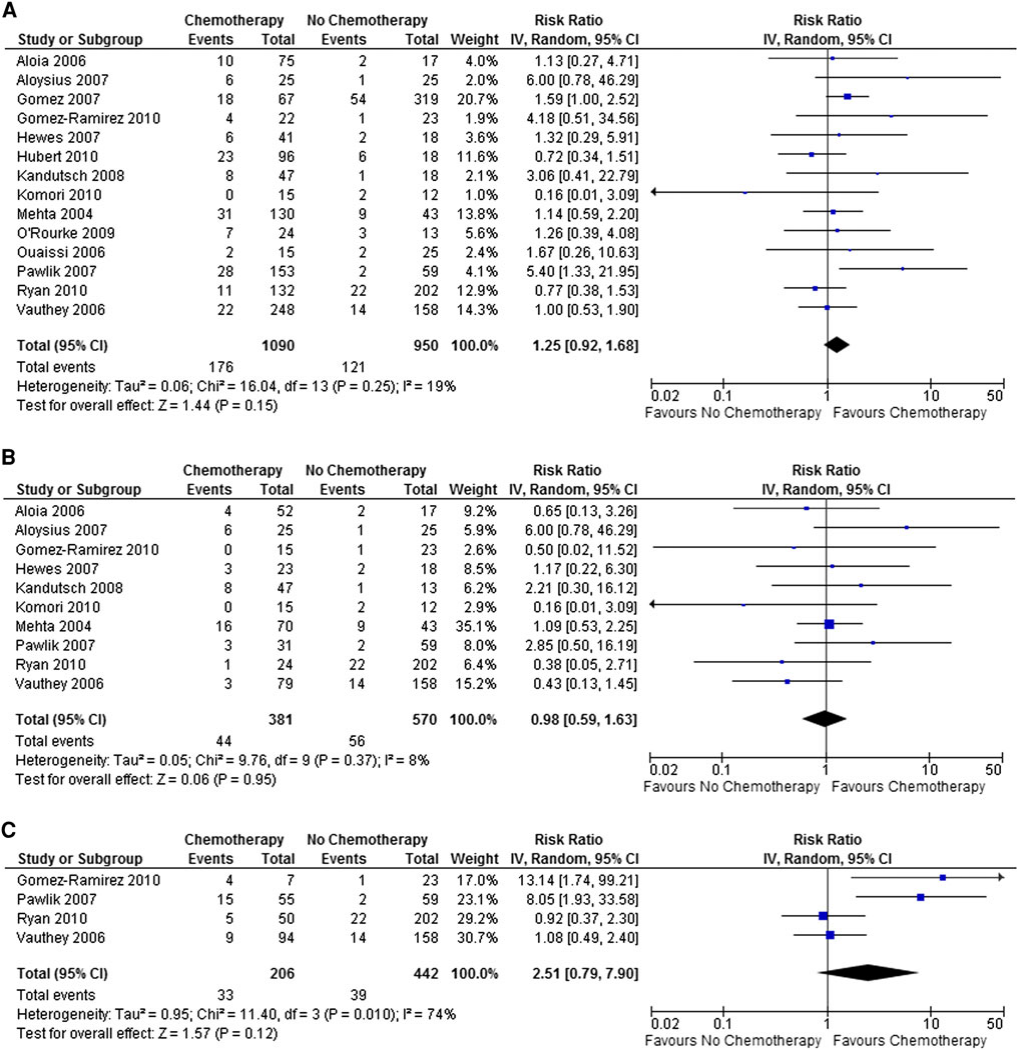
Risk of developing hepatic steatosis >30 % in patients treated with preoperative chemotherapy (**a**) and specifically in those receiving oxaliplatin-based regimens (**b**) or irinotecan-based regimens (**c**)

Thirteen studies reported the incidence of hepatic steatosis in 1,508 patients undergoing liver resection for colorectal liver metastases, 799 of whom had received preoperative chemotherapy. Overall, there was no association between the use of preoperative chemotherapy and the presence of hepatic steatosis (RR 1.25; 95 % confidence interval [CI] 0.99–1.57; *p* = 0.06). Similarly, no association could be demonstrated between the incidence of steatosis >30 % and the use of preoperative chemotherapy in 14 studies presenting data from 2,040 patients (RR 1.25; 95 % CI 0.92–1.68; *p* = 0.15; Fig. 2a).

It is increasingly recognized that the nature of chemotherapy-associated liver injury is regimen specific. In the case of oxaliplatin-based regimens, there was no association with the development of hepatic steatosis overall (RR 1.30; 95 % CI 0.85–2.00; *p* = 0.23) or steatosis >30 % (RR 0.98; 95 % CI 0.59–1.63; *p* = 0.95; Fig. 2b). Similarly, when the effects of irinotecan-based regimens were examined, it was not possible to demonstrate an increased incidence of steatosis >30 % (RR 2.51; 95 % CI 0.79–7.90; *p* = 0.12; Fig. 2c). This latter analysis included four studies, among which there was marked heterogeneity (*I* ^2^ = 74 %; *p* = 0.01), with the two smaller studies showing a markedly increased risk of steatosis >30 % in those receiving irinotecan-based chemotherapy, whereas the two larger studies demonstrated no such increase.^30^^–^ 33

One explanation for this heterogeneity in the included studies may be related to the manner in which hepatic steatosis is assessed: by review of hematoxylin and eosin-stained sections by an expert pathologist. In 2009 El-Badry et al. compared the grading of steatosis in 46 consecutive patients undergoing liver resection by four expert liver pathologists from three different countries. This study found that there was marked discrepancy among pathologists in the grading of steatosis, which grew more marked as the degree of steatosis worsened.^28^ A further study by Gawrieh et al.,^27^ has reported similar findings confirming the potential importance of this phenomenon.

Steatohepatitis is distinguished from simple steatosis by the presence of inflammatory infiltrates within the liver and ballooning degeneration of hepatocytes.^21^ Nonalcoholic steatohepatitis is most commonly associated with the presence of type 2 diabetes mellitus, obesity, and metabolic syndrome, meaning that the population prevalence of these conditions will directly affect the frequency of steatohepatitis in patients presenting for liver resection.^34^ Studies in patients undergoing bariatric surgery have suggested that in patients with a body mass index (BMI) of >35 kg/m^2^, the prevalence of steatohepatitis approaches almost 40 %.^35^

The presence of steatohepatitis is more worrying than simple steatosis when undertaking major liver resection, and its presence has been demonstrated to be associated with increased surgical morbidity and mortality after resection of colorectal liver metastases.^33^ It should be highlighted that in this study, all deaths in patients with steatohepatitis occurred in those who underwent combined resection and radiofrequency ablation. The significance of this is to emphasize that careful consideration needs to be given to safety when performing extensive procedures in patients with steatohepatitis.

**Fig. 3:**
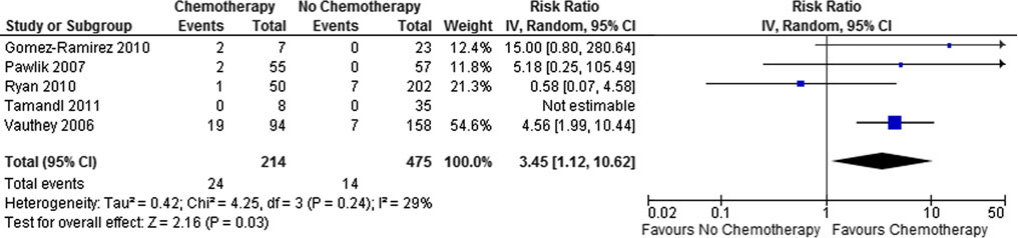
Irinotecan-based chemotherapy is associated with an increased risk of steatohepatitis

Overall, the use of preoperative chemotherapy was associated with a trend toward an increased incidence of steatohepatitis and was of borderline statistical significance (RR 1.89; 95 % CI 0.99–3.63; *p* = 0.05). If the analysis was limited to those receiving irinotecan-based regimens, however, there was a 3.45-fold increased risk of steatohepatitis when compared to those who were chemotherapy naive (95 % CI 1.12–10.62; *p* = 0.03; Fig. 3), giving a number needed to harm of 12 (95 % CI 7.8–26.5)—that is, 1 in every 12 patients treated with an irinotecan-based chemotherapy regimen would be expected to develop steatohepatitis as a result.

It can be seen that there is a moderate degree of heterogeneity in the included studies that arises from that of Ryan et al., who did not demonstrate any association between irinotecan-based chemotherapy and the development of steatohepatitis. This may in part be explained by the observation that the mean time interval between the cessation of chemotherapy and surgery in this study was 12 weeks as compared to 6 weeks in that of Vauthey et al., which demonstrated the strongest association between irinotecan use and steatohepatitis.^32^^,^ 33

The duration of chemotherapy administration is also a potential source of heterogeneity; however, this is only reported in two studies. In the study of Ryan et al.,^32^ patients typically received a mean of 7.5 cycles of chemotherapy, whereas in the study of Vauthey et al.,^33^ chemotherapy was administered for a median of 16 weeks. The different manners of reporting these make it difficult to make direct comparisons, although one cycle of chemotherapy typically lasts for 2 weeks, suggesting that both studies are broadly similar in this regard.

As already discussed, patient characteristics, particularly in regard to BMI and the prevalence of diabetes, may be significant sources of heterogeneity when comparing the prevalence of steatohepatitis between studies.^36^ In the studies of Gomez-Ramirez et al. 30 and Pawlik et al.,^31^ both of which reported a positive association between steatohepatitis and irinotecan, patients receiving irinotecan were more likely to have a higher mean BMI (29.3 vs. 26.2 and 28.1 vs. 26.6 kg/m^2^, respectively). In the study of Vauthey et al.,^33^ the incidence of steatohepatitis in patients with a BMI of >25 kg/m^2^ was nearly twice that in those with BMI of <25 kg/m^2^ (24.6 vs. 12.1 %). Multivariate analysis in the study of Ryan et al. 32 demonstrated that the only variable independently associated with steatohepatitis was a BMI of >30 kg/m^2^. None of the included studies performed prechemotherapy liver biopsies, and as such, it is impossible to truly determine what effect background steatosis has on the development of steatohepatitis after irinotecan treatment.

Oxaliplatin-based regimens were not associated with an increased risk of steatohepatitis (RR 1.17; 95 % CI 0.45–3.04; *p* = 0.75).

### Sinusoidal Injury

Until the advent of modern chemotherapeutics, sinusoidal obstruction syndrome was considered a rare phenomenon related to the ingestion of pyrrolizidine alkaloids.^37^ More recently, sinusoidal obstruction syndrome has been described in patients receiving myeloablative chemotherapy before bone marrow transplantation and latterly in the treatment of colorectal liver metastases.^38^ A key feature of sinusoidal obstruction syndrome is sinusoidal dilatation with associated hepatocyte atrophy. Later changes include the development of perisinusoidal fibrosis and nodular regenerative hyperplasia. Most commonly, sinusoidal dilatation is graded according to the method of Rubbia-Brandt et al. 22 (0 = absent, 1 = mild, 2 = moderate, 3 = severe), and a higher score is thought to reflect a more severe injury to the hepatic sinusoid.

**Fig. 4:**
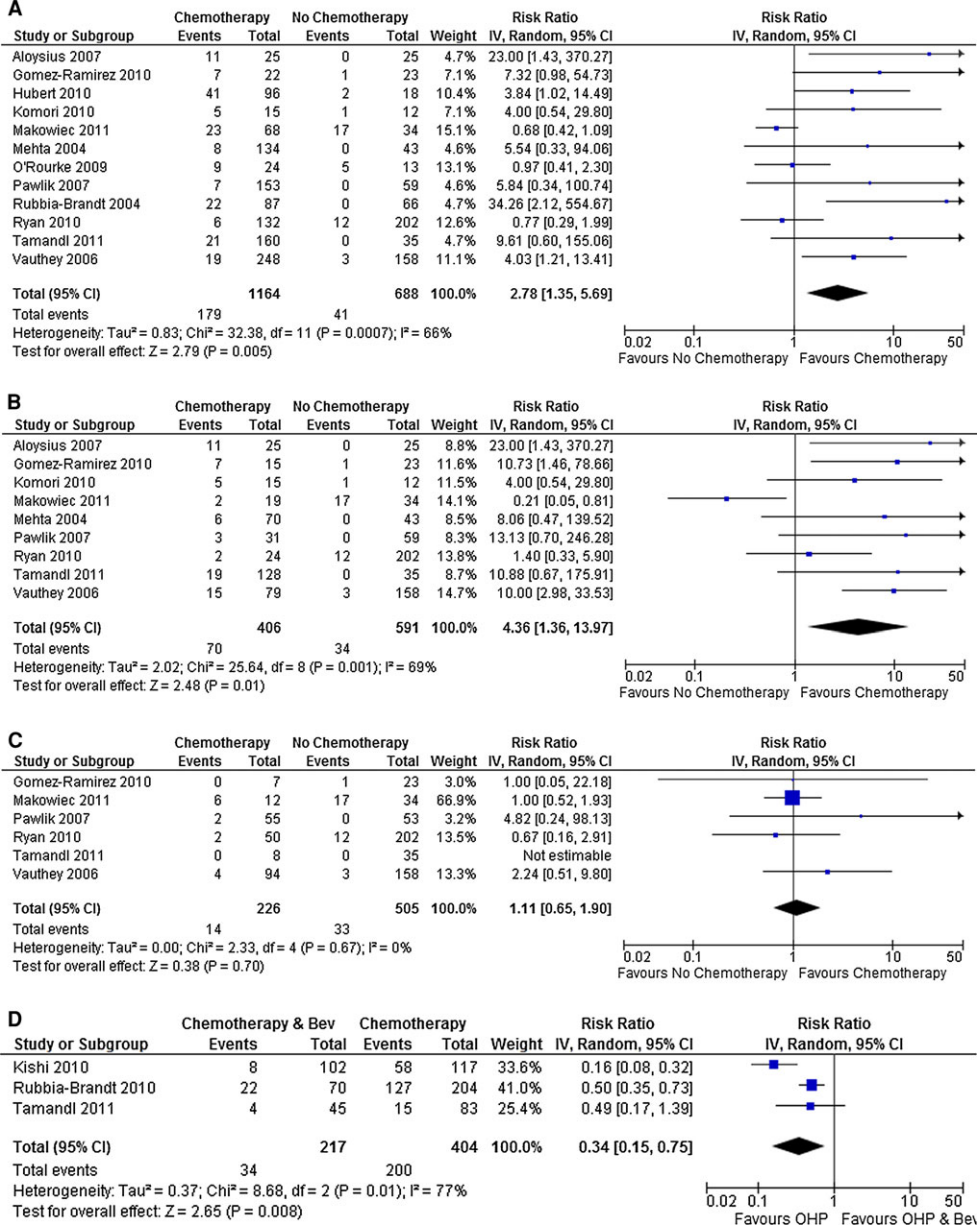
Risk of developing grade 2 or greater sinusoidal injury in patients treated with preoperative chemotherapy (**a**) and specifically with oxaliplatin-based regimens (**b**) or irinotecan-based regimens (**c**). The addition of bevacizumab to oxaliplatin-based chemotherapy reduces the risk of developing grade 2 or greater sinusoidal injury (**d**)

Eight studies reported the incidence of sinusoidal dilatation (grades 1–3) in a total of 871 patients, 633 of whom had received preoperative chemotherapy. The use of preoperative chemotherapy was associated with a 1.95-fold increased risk of sinusoidal dilatation (95 % CI 1.46–2.61; *p* < 0.00001). Grade 2 sinusoidal injury or greater is generally accepted as being a more accurate mark of sinusoidal injury and was reported in a total of 12 studies including a total of 1,852 patients.^22^,^38^ The use of preoperative chemotherapy was associated with a 2.78-fold increase in risk of grade 2 sinusoidal injury when compared to chemotherapy-naive controls (95 % CI 1.35–5.69; *p* = 0.005; Fig. 4a). However, there was a significant amount of heterogeneity in the included studies (*I* ^2^ = 66 %; *p* = 0.0007), again suggesting that the chemotherapy regimen may be important in determining who develops this pathology.

Six studies reported the incidence of sinusoidal dilatation in 333 patients receiving oxaliplatin-based chemotherapy as compared to 198 who were chemotherapy naive. Oxaliplatin-based regimens were found to be associated with a 2.22-fold increase in the risk of developing sinusoidal dilatation in the non-tumor-bearing liver (95 % CI 1.47–3.36; *p* = 0.0002). Similarly, a meta-analysis of nine studies reporting grade 2 or greater sinusoidal injury demonstrated that those receiving oxaliplatin-based regimens were at a 4.36-fold increased risk of this when compared to chemotherapy-naive control subjects (95 % CI 1.36–13.97; *p* = 0.01; Fig. 4b), with the number needed to harm being 8 (95 % CI 6.4–13.6). Surprisingly, there is a large degree of heterogeneity in this latter analysis (*I* ^2^ = 69 %; *p* = 0.001), which arises predominantly from the trial of Makowiec et al.,^41^ which reported an unusually high incidence of grade 2 sinusoidal injury in patients who were chemotherapy naive (17 of 34), which is much greater than that reported in the other studies.

In a multivariate analysis of factors associated with sinusoidal dilatation of all grades, Nakano et al.^39^ identified receiving >six cycles of oxaliplatin-based chemotherapy to be an independent risk factor (RR 3.2; *p* = 0.048). In contrast, Tamandl et al.^40^ did not demonstrate any association between the number of cycles of chemotherapy and the development of grade 2 or greater sinusoidal dilatation on univariate analysis (hazard ratio 0.70; *p* = 0.502). The number of cycles of chemotherapy administered was reported in seven out of nine studies included in the meta-analysis in Fig. 4b and is summarized in Table 2. Differences in this regard did not explain the absence of association between oxaliplatin-based chemotherapy and grade 2 sinusoidal dilatation reported in the studies of Makowiec et al.^41^ and Ryan et al.^32^

**Table 2:** Chemotherapy details in studies included in studies analyzing the risk of grade 2 or greater sinusoidal dilatation after oxaliplatin-based chemotherapy

Study	Years	Interval between chemotherapy and surgery	Number of chemotherapy cycles
Aloysius et al.^69^	2007	–	Median 6
Gomez-Ramirez et al.^30^	2010	4–6 weeks	–
Komori et al.^42^	2010	Mean 37 days	Mean 7.7
Makowiec et al.^41^	2011	26 % Patients > 6 months	Median 6
Mehta et al.^26^	2004	–	–
Pawlik et al.^31^	2007	–	65 % Less than 12 weeks duration
Ryan et al.^32^	2010	Mean 15 weeks	Mean 8.6
Tamandl et al.^40^	2011	–	Median 6
Vauthey et al.^33^	2006	Median 6.4 weeks	Median 12-week duration

The time interval between cessation of chemotherapy and liver resection were reported in five of nine studies from the meta-analysis in Fig. 4b and are summarized in Table 2. It can be seen that three of these studies report a time interval in the order of 4–6 weeks, all of which report a positive association between oxaliplatin-based chemotherapy and grade 2 sinusoidal dilatation.^30^,^42^,^43^ In contrast, the two studies that failed to demonstrate such an association had longer time intervals, with Ryan et al.^32^ reporting a mean of 15 weeks, and in the study of Makowiec et al.,^41^ 26 % of patient had a time interval of over 6 months. These findings might suggest that the changes of sinusoidal obstruction syndrome are at least partly reversible with time, although there is insufficient evidence to prove this.

No association could be demonstrated between the use of irinotecan-based chemotherapy regimens and the development of grade 2 or greater sinusoidal dilatation (RR 1.11; 95 % CI 0.65–1.90; *p* = 0.70; Fig. 4c).

In addition to sinusoidal dilatation, more severe sinusoidal obstruction syndrome is associated with features such as nodular regenerative hyperplasia, peliosis, and parenchymal extinction.^44^ The presence of these features was assessed in the series of Rubbia-Brandt et al.,^44^ who found that patients treated with oxaliplatin-based chemotherapy demonstrated an increased incidence of nodular regenerative hyperplasia (58 vs. 0 %) compared to chemotherapy-naive controls. Peliosis was also more common in patients treated with oxaliplatin-based chemotherapy, and its presence was linked to the severity of sinusoidal dilatation being present in 30 % of patients with grade 3 dilatation as compared to 1 % in those with a grades 1 or 2 injury.^44^ The association between oxaliplatin-based chemotherapy and nodular regenerative hyperplasia was not confirmed in the two other studies that reported this outcome, those of Komori et al.^42^ and Ryan et al.,^32^ although the number of patients treated with oxaliplatin-based chemotherapy in both these series was small (15 and 24, respectively).

Bevacizumab is a monoclonal antibody directed against VEGF-A, a potent mediator of angiogenesis. A number of publications have recently suggested that the addition of bevacizumab to conventional oxaliplatin-based regimens may reduce the incidence of sinusoidal obstruction syndrome.^44^^–^ 46 Of the two studies that reported all grades of sinusoidal dilatation in 115 patients receiving oxaliplatin-based chemotherapy alongside bevacizumab, there was no difference in risk when compared to 287 patients receiving oxaliplatin-based chemotherapy alone (RR 0.86; 95 % CI 0.72–1.04; *p* = 0.31). Three studies examined the incidence of grade 2 or greater sinusoidal injury, demonstrating that the addition of bevacizumab to conventional oxaliplatin-based regimens reduces the risk of injury by almost threefold (RR 0.34; 95 % CI 0.15–0.75; *p* = 0.008; Fig. 4d). Calculating the number needed to treat reveals that the addition of bevacizumab to oxaliplatin-based chemotherapy would be expected to prevent the development of sinusoidal obstruction syndrome in one out of every three patients (95 % CI 2.5–3.7).

## Discussion

The role of chemotherapy in the preoperative management of patients with colorectal liver metastases is one of the most keenly debated topics among those treating this condition.^43^,^47^,^48^ It is universally accepted that patients with inoperable disease should be treated, where possible, with aggressive chemotherapy with a view to downstaging disease such that curative surgery can be offered.^11^,^12^,^47^,^49^ It has been demonstrated in several series that overall survival in this patient group compares favorably to those able to undergo surgery from the outset.^13^,^50^,^51^

What remains much less clear is what role, if any, preoperative chemotherapy has to play in the management of patients presenting with operable liver only metastases. Although the EPOC trial attempted to answer this question, it is not clear from this study whether the benefits seen in terms of progression-free survival were attributable to preoperative therapy, adjuvant therapy, or a combination of both.^14^ The major cause for concern when chemotherapy is used in this context is the potential effects on the hepatic parenchyma and the subsequent implications this may have on surgical morbidity and mortality.^17^,^18^ In the EPOC study, it was demonstrated that the incidence of postoperative complications was significantly increased in the FOLFOX arm as compared to those who underwent surgery alone (25 vs. 16 %; *p* = 0.04), although there was no difference in mortality.^14^

The meta-analysis of published studies we have performed has demonstrated that the nature of the parenchymal injury that results from preoperative chemotherapy cannot be generalized as a global effect but rather is a regimen-specific phenomenon—that is, irinotecan-based regimens are associated with steatohepatitis whereas oxaliplatin-based regimens are associated with sinusoidal obstruction. It is also noteworthy that the addition of bevacizumab to oxaliplatin-based regimens appears to reduce the severity of oxaliplatin-induced sinusoidal obstruction syndrome, although the number of patients included in the three studies reporting this is small, and larger studies are needed to prove the association. Despite the widespread clinical use of the anti-EGFR monoclonal antibodies cetuximab and panitumumab, there are no published data regarding their effect on chemotherapy-induced liver injury. However, they are usually provided in combination. This is an area that needs clarification.

It is perceived that an increased risk of morbidity in these patients arises from injury to the hepatic parenchyma, and this view is supported by a number of studies that have demonstrated the negative impact of parenchymal disease on surgical outcome in those having major resection.^52^^–^ 55 Before embarking on a major hepatectomy, it is routine to make an evaluation of the liver either radiologically (e.g., steatosis, splenomegaly) or by using specific tests of hepatic functional reserve, such as the indocyanine green retention rate, the MEGX test, or the LiMAx test.^56^^–^ 59 Krieger et al.^60^ demonstrated that patients who received preoperative chemotherapy were more likely to have a greater indocyanine green retention at 15 min as compared to those who are chemotherapy naive (7.3 vs. 3.5 %; *p* < 0.001). A multivariate analysis performed by Nakano et al.^39^ demonstrated that a preoperative indocyanine green retention rate of >10 % was an independent predictor of the presence of sinusoidal injury (RR 4.02; 95 % CI 1.26–12.88; *p* = 0.019). When it is determined that an individual patient is at high risk of chemotherapy-induced liver injury, it may be necessary to modify the planned surgical procedure to spare more of the liver parenchyma or to use measures such as portal vein embolization to increase the size of the planned future hepatic remnant, thereby minimizing the risk of postoperative liver failure.^61^ It has been suggested that hypertrophy of the future liver remnant after portal vein embolization may be impaired in patients who have received preoperative chemotherapy, although this is disputed by others, and further clarification is needed on this subject.^62^^–^ 64

Identifying patients at particular risk of developing a parenchymal injury after preoperative chemotherapy has proven difficult. Despite the logical belief that prolonged chemotherapy exposure is related to an increased incidence of injury, the evidence in relation to this is difficult to interpret, with conflicting results being reported, particularly in regard to oxaliplatin-induced sinusoidal obstruction syndrome, suggesting that the story is perhaps more complicated.^39^,^40^ It may be that patients with pre-existing liver disease are at an increased risk of parenchymal injury, although the absence of a prechemotherapy liver biopsy makes it difficult to ascertain to which patient groups specifically this might apply. It is increasingly recognized that pharmacogenomics can play a key role in determining the susceptibility of the individual to the toxic effects of chemotherapy; for example, patients with mutations in the *UGT1A1* gene have been found to be at increased risk of systemic toxicity from irinotecan.^65^ Similarly, oxaliplatin toxicity is affected by mutations in genes involved in DNA damage repair and conjugation of its metabolites to glutathione.^66^ Whether genetic polymorphisms in these or other genes are able to identify a cohort of patients at increased risk of chemotherapy-induced parenchymal injury is not known, but this area may well be worthy of further exploration.

In conclusion, preoperative chemotherapy is associated with regimen-specific liver injury. The presence of such an injury may have a negative impact on the functional reserve of the liver, thereby increasing the risk of surgical morbidity and mortality. This should be borne in mind when planning multimodal treatment for patients with colorectal liver metastases.
